# Mortality, disease and associated antimicrobial use in commercial small-scale chicken flocks in the Mekong Delta of Vietnam

**DOI:** 10.1016/j.prevetmed.2019.02.005

**Published:** 2019-04-01

**Authors:** Juan Carrique-Mas, Nguyen Thi Bich Van, Nguyen Van Cuong, Bao Dinh Truong, Bach Tuan Kiet, Pham Thi Huyen Thanh, Nguyen Ngoc Lon, Vu Thi Quynh Giao, Vo Be Hien, Pawin Padungtod, Marc Choisy, Erry Setyawan, Jonathan Rushton, Guy Thwaites

**Affiliations:** a Oxford University Clinical Research Unit, Vietnam; b Centre for Tropical Medicine and Global Health, Nuffield Department of Medicine, Oxford University, Oxford, United Kingdom; c Faculty of Animal Science and Veterinary Medicine, Nong Lam University, Ho Chi Minh, Vietnam; d Sub-Department of Animal Health, Cao Lanh, Dong Thap, Vietnam; e Emergency Center for Transboundary Animal Diseases, Food and Agriculture Organization of the United Nations, Green One UN House Building, 304 Kim Ma, Hanoi, Vietnam; f MIVEGEC, IRD, CNRS, University of Montpellier, France; g Food and Agriculture Organization of the United Nations, Jakarta, Indonesia; h Institute of Infection and Global Health, University of Liverpool, United Kingdom

**Keywords:** Mortality, Disease, Antimicrobial use, Poultry, Chickens, Vietnam

## Abstract

Raising chickens in small-scale flocks following all-in-all-out management is common in the Mekong Delta of Vietnam. These flocks represent an intermediate category between backyard and intensive (industrial) farming systems. However, little is known about the occurrence and burden of disease and/or mortality in such flocks, and their potential association with antimicrobial usage (AMU). We investigated mortality, disease and weekly antimicrobial use (AMU) in 124 cycles of meat chicken flocks raised in 88 farms in the Mekong Delta of Vietnam (with a median cycle duration of 18 weeks [inter-quartile range IQR 17–20]). We visited each farm 4 times per cycle to review data collected weekly by the farmers on clinical signs, mortality, and AMU. The overall probability of disease and AMU were 0.31 (95% CI 0.29–0.32) and 0.26 (95% CI 0.24–0.28), respectively. The average weekly incidence of mortality was 2.6 (95% CI 2.2–3.0) per 100 birds. Both the probabilities of a flock experiencing disease and mortality, as well as of using antimicrobials decreased with the flock’s age. However, mortality peaked at the 5–10 week period. The only significant explanatory factors associated with presence of disease was the stage of production ≥5 weeks (protective) (OR ≤ 0.51). Factors independently associated with AMU (*p* < 0.05) were: (1) Number of chickens (log) (OR=1.46), (2) Stage of production ≥5 weeks (OR≤0.67) (protective), (3) Cao Lanh district (OR=2.23), (4) Density of veterinary drug shops at commune level (log) (OR=1.58), and (5) Disease in flocks (OR=1.80). Factors independently associated with overall increased weekly incidence of mortality (*p* < 0.05) were: (1) High level of education attainment (secondary education or higher) (Hazard rate Ratio [HR]=1.70), (2) number of chickens (log) (HR=1.39), and (3) Stage of production >5 weeks (HR≤2.14). In flocks reporting disease, AMU significantly reduced the incidence of mortality (HR=0.90). These results confirm an exceptionally high mortality in chicken flocks in the area, jeopardizing the profitability and sustainability of these small-scale farming systems. The data also suggest an association between nearby access to antimicrobials and AMU, and a high correlation of AMU over consecutive cycles. The atomized farming landscape of the Mekong Delta, the high incidence of disease and mortality, and the unrestricted and easy access to antimicrobials present major challenges to the implementation of policies aimed at AMU reductions.

## Highlights

• High mortality (2.6 chickens/100 chickens/week) in small-scale Mekong Delta flocks.
• Disease most common in the brooding period; mortality peaks in the 5–10 week period.
• Antimicrobials use (AMU) most common in the early (‘brooding’) period.
• Farmers tend to repeat AMU behavior over consecutive flock cycles.
• AMU was associated with the density of veterinary drug shops.

## 1 Introduction

With over 100 million tons produced per year (2014) chicken meat is the second most common animal food commodity worldwide (FAO, 2017). In low- and middle-income countries, chickens are often raised in backyard and small-scale and flocks, supporting rural livelihoods by providing animal protein and nutrients (meat and eggs), as well as manure and feather bio-products. In many countries chicken meat is also central to festivities and traditional ceremonies (Alders and Pym, 2009; FAO, 2010). Therefore, high levels of disease and mortality in small-scale farms pose major constraints to the livelihoods of large numbers of poor people worldwide, and infectious diseases are thought to be responsible to a large extent (Bell, 2009). Over recent years, more and more farms in the Mekong Delta have been upgrading their production capacity, transitioning from ‘backyard’ to confined housing and flock management using all-in-all-out principles. Much of the published research on poultry diseases in southeast Asia has consisted on the detection and characterization of single bacterial and viral pathogens (Jonas et al., 2001; Eagles et al., 2009; Chukiatsiri et al., 2012). In Vietnam, research has overwhelmingly focused on Highly Pathogenic Avian Influenza (HPAI) (Lee et al., 2015; Nguyen et al., 2017), due to its high pathogenicity in poultry, and its pandemic potential. Although HPAI is still endemic in the Mekong Delta of Vietnam, large outbreaks of the disease are now less common compared with the 2003–2006 period, when the HPAI H5N1 epidemic was first reported (Anon, 2018; FAO, 2018; Meyer et al., 2018). In addition to HPAI, several viral poultry diseases, such as Newcastle Disease (Choi et al., 2014), Infectious Bursal Disease (IBD), and Infectious Bronchitis (IB) (de Witt et al., 2010) are all suspected to be widely circulating in Vietnam, and therefore vaccination programmes largely focus on these diseases (Bui et al., 2001). However, no data on circulation/incidence of these viral diseases, as well as major bacterial diseases and coccidiosis in the area are available.

Antimicrobial use (AMU) in animal production is a key driver of antimicrobial resistance (AMR) worldwide (O’Neill, 2015). It has been estimated that, worldwide, on average, 148 mg of antimicrobial active principle are used to raise 1 kg of live chicken, closely following antimicrobial use in pig production (172 mg) (Van Boeckel et al., 2015). In the Mekong Delta region of Vietnam high levels of AMU in chicken production have been reported (˜260 mg kg of chicken, excluding medicated feed) (Carrique-Mas et al., 2014; Trung et al., 2015). These quantities are, in part, due to the widespread circulation of infectious diseases, which in turn is associated with deficient levels of sanitation and health management – often termed ‘poor farm biosecurity’ (Hong Hanh et al., 2007b). In 2015 there were 277 million chicken heads in Vietnam, ˜20% of which were in the Mekong Delta (FAO, 2017). The number of households engaged in small-scale poultry production in the country is estimated in about 8 million, with an average flock size of ˜32 birds (Burgos et al., 2007). Small-scale poultry production plays an important role in rural areas, contributing to 19% of household income (Desvaux et al., 2008). In spite of the importance of small-scale chicken farming in Southeast Asia, there is limited information on disease patterns and mortality in these systems. To address this critical gap, we investigated a large sample of chicken farms with the following aims: (1) to quantify mortality; (2) to characterise disease patterns; and (3) to investigate associations between AMU, disease and mortality in flocks. The knowledge on disease and associated mortality in smallholder poultry flocks is an important and necessary step to improve farm management and adopt effective control measures to improve farm productivity and help reduce the farmer’s reliance on antimicrobials.

## 2 Materials and methods

### 2.1 Study location and farm recruitment

This study was carried out on farms raising chickens for meat with a flock capacity of >100 birds (case definition) in the districts of Cao Lanh and Thap Muoi within Dong Thap province (Mekong Delta region of Vietnam), as part as the baseline phase of a research project (Carrique-Mas and Rushton, 2017). These small-scale commercial flocks lie between ‘backyard’ flocks and intensively managed ‘industrial’ systems. These flocks roughly correspond to FAO Sectors 2 and 3 (between 50 and 2000 birds, with feed and water supplied to the birds) (FAO, 2010). Meat chicken flocks are typically based on slow-growing local breeds (4–5 months to reach a market weight of 1.6-2.0kg), raised as single age and confined in a dedicated house/pen. The chickens are kept at ambient temperature, except for the brooding period (first 4 weeks), where chicks receive additional heating. However, in some cases, chickens may have some access to grazing areas within the farm. In some instances, farmers may purchase day-old chicks from several sources over the first few weeks, and birds are often sold over a period of 1–4 weeks. All feed- and water-dispensation is manual, with flocks being predominantly raised on commercial feed. A total of 207 farmers randomly selected from the census were contacted by letter by the veterinary authorities (sub-Department of Animal Health and Production of Dong Thap, SDAHP). A meeting was held with 199 attending farmers (96%), where the project aims and methods were presented. Farmers where asked to contact project staff as soon as they restocked with day-old chicks. From each study farm, chicken flocks (defined as a group of birds raised together in the same building) that met the case definition and had completed at least one full production cycle over the time frame of the study were included (study flocks).

**Fig. 1:**
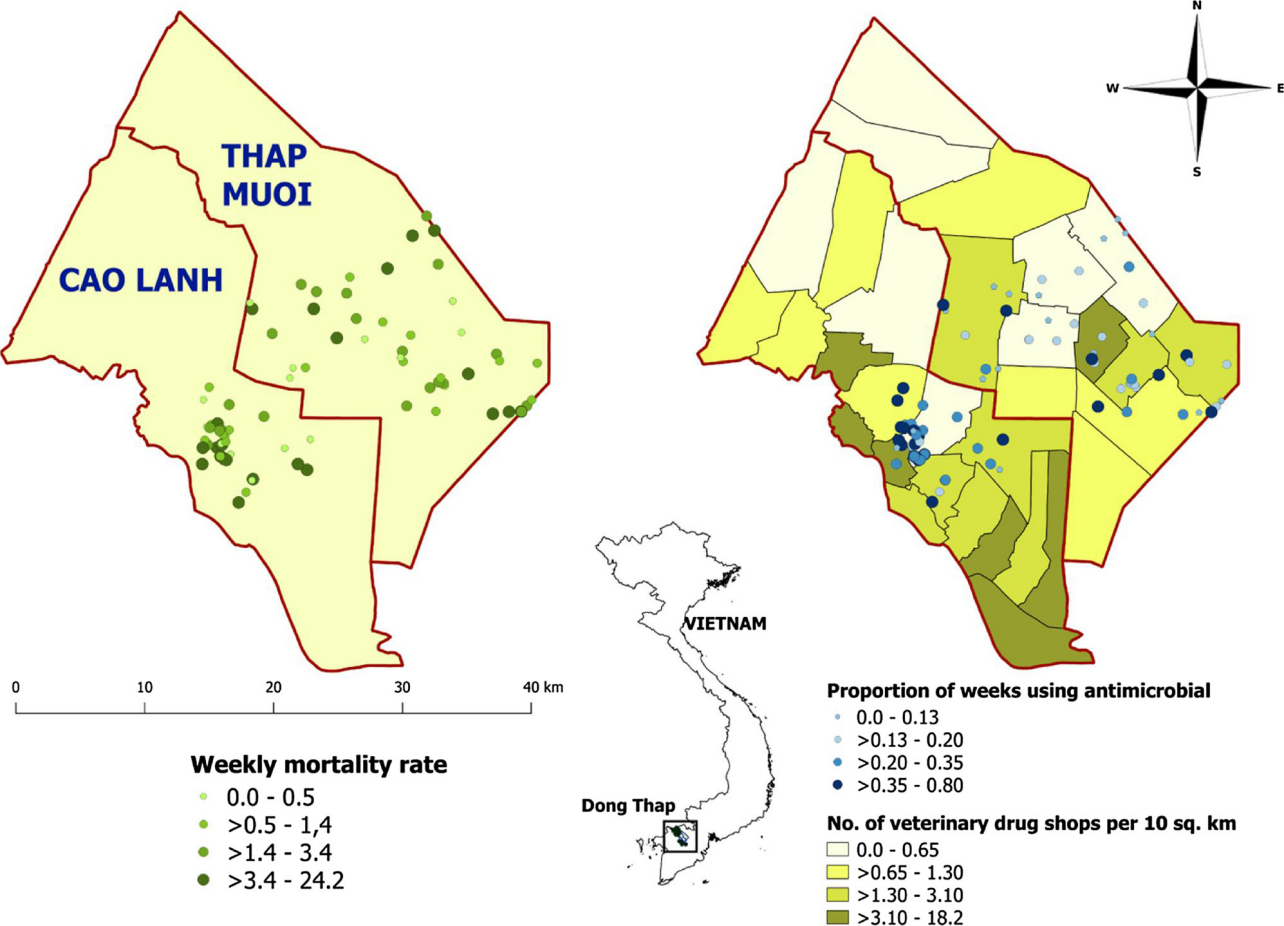
Location of study farms (n = 88) in the two study districts (Cao Lanh and Thap Muoi) within Dong Thap province. The average weekly incidence of mortality (per 100 birds), as well as the proportion of weeks that farmers used antimicrobials, and the density of veterinary drug shops are displayed.

Of 106 farmers that met the case definition that planned to restock within 4 months of the meeting, 88 agreed to participate in the study (84% participation). These 88 farms were investigated over a total of 124 fully completed production flock cycles from October 2016 to March 2018 (54 farms over 1 cycle, 32 over 2, and 2 over 3 consecutive cycles). Farm visits were carried out by veterinarians affiliated to the SDAHP. Farm location is shown in Fig. 1.

### 2.2 Data collection

Farmers were provided with a notebook laid out as two A4 sides by week, and were instructed to note down the following information related to their flock: (1) Movements of chickens in and out of their farm (i.e. numbers of birds bought, sold and dead on farm); (2) Any observed clinical signs, including: malaise (ruffled feathers, prostration), signs of respiratory infection (sneezing, coughing, wheezing, nasal secretion), enteric infection (diarrhoea), signs of central nervous system (CNS) disorder (ataxia, torticollis, circling) or other signs (i.e. lameness); (3) Use of health-supporting products (including vaccines). Farmers were instructed to keep bottles and containers of all health-supporting products used on their flocks. Farms were visited four times during each production cycle to review the data. On the first visit, generic data on the farmer, the farm and the chicken house were collected. On this first visit farmers were also trained by project veterinarians to recognize the main clinical signs, supported by a Vietnamese text book on poultry diseases that contains a description and visual images of the most common signs of chicken flocks where appropriate. This training was repeated several times on subsequent visits to the farms. Data on flock-related variables were collected on subsequent visits. Visiting veterinarians reviewed the labels of all commercial products given to the chickens and determined which products contained antibacterial antimicrobial active ingredients.

### 2.3 Statistical analyses

‘Disease’ was defined for a flock on a given week when signs of disease were observed in at least 5% of the birds in the flock. The probability of ‘Disease’, and the probability of using antimicrobials was computed for each week of the production cycle, with the total number of flocks observed on any given week taken as the denominator. The ‘weekly (cumulative) incidence of mortality’ was calculated for all study flocks by dividing the total number of chickens dying each week by the total number of chickens present on farms at the beginning of each week. Any chickens purchased halfway through the week were included in the denominator for the calculation of the following week period. The flock cycle (cumulative) incidence of mortality was calculated for each flock cycle by dividing the total number of birds dying from restocking to sale, divided by the total size of the flock at restocking. The weekly incidence of mortality was modelled using a Poisson model, with ‘Farm’ included as a random effect, and the size of the flock at the beginning of the week (log) as the offset. The association between farmer, farm, and flock characteristics (outcomes) and the variables ‘Disease’ (Yes/No)’ and ‘Antimicrobial Use (AMU)’ (Yes/No) (responses) were investigated by building multivariable logistic regression models, with ‘Farm’ modelled as a random effect. The following independent variables were investigated: Farmer-related: (1) Farmer/farm owner’s gender, (2) Age of farm owner (Years) (log), (3) Highest level of education attainment of farmer/farm owner, (4) Experience in chicken farming (Years) (log); Farm-related: (5) Type of chicken house, (6) Presence of chickens other than the target flock in the farm, (7) Presence of other poultry species other than chickens; Flock-related: (8) Number of chickens, (9) Week of production; Geographical variables: (10) District (Cao Lanh/Thap Muoi), (11) Number of chickens per km^2^ by commune, and (12) Number of veterinary drug shops per km^2^ by commune. Variables were ranked by their degree of significance, and were included in the models using a stepwise forward approach, starting with the ones with the lowest p-value obtained from the likelihood ratio test (LRT) comparing a model with and without the variable. The variables The variables ‘Disease’ and ‘Mortality’ (Yes/No) were investigated in the model with AMU as response variable to investigate to what extent AMU was a function of health events on farm. Variables with *p* ≤0.05 from the LRT were retained in final multivariable models. The Intra-cluster Correlation Coefficient (ICC) was calculated for the two final multivariable logistics models to investigate the percent of the total variation associated with the clusters (farms). The potential impact of AMU on the weekly incidence of mortality given disease was investigated by fitting a model on a subset of data corresponding to weeks where disease was reported, with weekly mortality as response, and the variables AMU (Yes/No) and clinical signs reported (respiratory, diarrhoea, CNS and malaise) as explanatory variables. The correlation between AMU in weeks with and without disease, as well as the correlation between AMU over subsequent cycles of production was estimated using the Pearson correlation coefficient. All statistical analyses were performed using the lme4 and MASS packages within R statistical software (http://www.r-project.org).

### 2.4 Ethics

This study was part of the ViParc project, which was granted ethics approval by the Oxford Tropical Research Ethics Committee (OXTREC) (Ref. 5121/16) and by the local authorities (People’s Committed of Dong Thap province) (May 2016).

## 3 Results

### 3.1 Study farms

The median flock size at restocking was 303 birds [inter-quartile range (IQR) 202–500]. The unadjusted prevalence of disease and/or mortality, AMU and the average mortality (per 100 birds) (per week) by levels of the variables investigated are shown in Table 1. The median duration of one production cycle was 18 [IQR 17–20] weeks. Most (81.8%) flocks were raised on houses/pens on solid ground, whereas others were housed on stilts, either over a canal (8.8%) or on the ground (5.7%). One flock was raised on two types of housing: solid house during the brooding period, and then transferred to a stilted house over a water canal during the grow-out period. A total of 44.3% farms were raising domestic ducks, 12.5% Muscovy ducks, 19.3% pigs and 2.3% cattle at the beginning of the study (Table 2)

**Table 1:** Unadjusted weekly probability of disease and/or mortality and antimicrobial use, and weekly incidence of mortality (per 100 birds) by study variables in chicken flocks for 124 cycles of production (Dong Thap, Mekong Delta, Vietnam).

	No. farms (*flocks) (No. weeks)	Disease (Y/N)		Weekly incidence of mortality (per 100 birds)		Antimicrobial use (Y/N)	
		Prop.	95% CI	Mean	95% CI	Prop.	95% CI
Farmer’s gender							
*Male*	11 (337)	0.32	0.30-0.34	2.60	2.14-3.05	0.27	0.25-0.29
*Female*	77 (1890)	0.23	0.19-0.27	2.48	2.03-2.94	0.21	0.16-0.25
Farmer's age							
*Up to 45*	44 (979)	0.31	0.28-0.38	2.90	2.22-3.58	0.27	0.24-0.30
*Over 45*	44 (1248)	0.30	0.28-0.33	2.33	1.83-2.83	0.26	0.23-0.28
Farmer's highest education attainment							
*Primary school*	22 (638)	0.25	0.21-0.28	1.56	1.02-2.11	0.27	0.24-0.31
*Secondary school*	36 (904)	0.32	0.29-0.35	2.35	1.76-2.93	0.26	0.23-0.29
*High school*	25 (544)	0.34	0.30-0.38	3.62	2.58-4.67	0.28	0.24-0.32
*Post high school*	5 (141)	0.35	0.28-0.43	4.67	2.37-6.98	0.20	0.13-0.26
Farmer's experience in chicken farming (years)							
*0-1.5*	20 (506)	0.28	0.24-0.32	2.96	2.02-3.91	0.25	0.22-0.29
*1.6-2.3*	32 (745)	0.31	0.28-0.34	2.71	1.94-3.48	0.27	0.24-0.30
*2.4-3.5*	21 (595)	0.34	0.30-0.38	2.22	1.57-2.87	0.25	0.21-0.28
*3.6-11.0*	15 (381)	0.28	0.24-0.33	2.39	1.46-3.33	0.28	0.24-0.33
Type of chicken house*							
*Solid ground*	72 (1873)	0.32	0.30-0.34	2.61	2.15-3.62	0.26	0.24-0.28
*Stilts on ground*	5 (79)	0.14	0.06-0.22	2.16	0.18-4.13	0.30	0.20-0.40
*Stilts on water*	10 (265)	0.27	0.22-0.33	2.21	1.36-3.06	0.26	0.21-0.31
*Solid and stilts*	1 (10)	0.30	0.02-0.58	10.52	0.0-30.0	0.50	0.19-0.81
Presence of chickens other than the target flock*							
*No*	57 (1015)	0.28	0.25-0.30	2.76	2.09-3.44	0.26	0.24-0.29
*Yes*	67 (1212)	0.33	0.30-0.36	2.43	1.93-2.93	0.26	0.24-0.29
Presence of non-chicken poultry species*							
No	55 (839)	0.30	0.27-0.33	2.78	2.18-3.38	0.27	0.24-0.30
Yes	69 (1389)	0.31	0.28-0.34	2.35	1.80-2.89	0.26	0.23-0.28
No. chickens restocked*							
*100-199*	22 (372)	0.20	0.16-0.24	1.82	1.25-2.39	0.23	0.19-0.27
*200-299*	30 (527)	0.28	0.24-0.32	2.02	1.22-2.83	0.24	0.21-0.28
*300-499*	38 (692)	0.31	0.28-0.35	3.40	2.52-4.28	0.26	0.23-0.29
*500+*	34 (636)	0.38	0.34-0.42	2.60	1.83-3.37	0.30	0.27-0.34
Week of production (age of flock)*							
*1-5*	124 (494)	0.48	0.44-0.53	2.42	1.95-2.88	0.39	0.33-0.42
*>5-10*	124 (607)	0.36	0.32-0.39	3.66	2.62-4.70	0.29	0.25-0.33
*>10-14*	116 (457)	0.22	0.18-0.25	1.96	1.18-2.74	0.21	0.17-0.24
*>14-26*	111 (545)	0.09	0.06-0.11	2.09	1.22-2.95	0.05	0.03-0.06
District							
*Thap Muoi*	46 (1282)	0.29	0.26-0.31	2.46	1.98-2.93	0.20	0.18-0.22
*Cao Lanh*	42 (945)	0.33	0.30-0.36	2.75	2.03-3.46	0.35	0.32-0.38
Commune density of chickens (per km^2^)*							
*1-320*	46 (1119)	0.28	0.25-0.31	2.68	2.12-3.25	0.21	0.18-0.23
*>320*	42 (1028)	0.33	0.30-0.36	2.46	1.87-3.05	0.33	0.30-0.358
*0-1*	50 (1226)	0.29	0.26-0.32	2.78	2.21-2.35	0.28	2.21-3.35
Commune density of veterinary drug shops (per 10km^2^)							
*>1*	38 (1001)	0.31	0.29-0.34	2.34	1.76-2.92	0.24	1.76-2.92

**Table 2:** Risk factors for mortality/disease and antimicrobial use (random effects logistic regression models) and mortality (Poisson models).

	Disease (Y/N)	AMU (Yes/No)		Weekly incidence of mortality (overall)		Weekly incidence of mortality (in weeks reporting disease)
	Univariable	Univariable	Multivariable††	Univariable	Multivariable†††	Multivariable††††
	OR (p-value)	OR (p-value)	OR [95% CI]	HR (p-value)	HR [95% CI]	HR [95% CI]
Gender (female)	0.72 (0.210)	0.67 (0.120)		1.14 (0.688)		
Farmer’s age (years) (log)	1.03 (0.943)	0.96 (0.900)		0.90 (0.797)		
High school or higher education	1.37 (0.151)	1.07 (0.730)		1.69 (0.030)	1.70* [1.04-2.80]	1.58* [1.03-2.44]
Experience in chicken farming (years) (log)	1.04 (0.841)	1.10 (0.540)		1.24 (0.279)		
Solid ground chicken house (Ref. Stilts)	1.52 (0.136)	0.95 (0.840)		0.76 (0.394)		
Other chicken flock/s	1.30 (0.192)	1.01 (0.970)		0.90 (0.660)		
Other (non-chicken) poultry	1.04 (0.842)					
No. chickens (log)	1.17 (0.236)	1.48 **	1.46‡‡ [0.98-2.17]	1.04*	1.39*** [1.31-1.47]	0.89*** [0.84-0.94]
Week of production (Ref. 1-4)						
5-10	0.51***	0.39 ***	0.67** [0.51-0.90]	1.98***	2.14*** [2.06-2.22]	2.87*** [2.74-3.0]
>10-14	0.21***	0.22***	0.42*** [0.30-0.59]	1.31***	1.55*** [1.46-1.64]	3.15*** [2.95-3.38]
>14-26	0.05***	0.03***	0.06*** [0.04-0.10]	1.33***	1.72*** [1.31-1.47]	7.52*** [6.93-8.17]
Cao Lanh district	1.27 (0.241)	2.16***	2.23** [1.25-3.96]	0.86 (0.511)		
Log(Density of veterinary drug shops)	0.79 (0.121)	1.12 (0.367)	1.58** [1.13-2.20]	0.85 (0.350)		
Log(Density of chickens)	1.08 (0.436)	1.39 (<0.001)		0.85 (0.121)		
Disease (Yes/No)	–	4.28 (<0.001)	1.80* [1.02-3.18]	–		
Mortality (Yes/No)		4.64 (<0.001)				
AMU	–	–		–		0.90*** [0.86-0.94]

HR=Hazard rate Ratio; *p<0.05; **p<0.01; ***p<0.001; ‡p=0.069; ‡‡p=0.061; ^†^Model intercept: -0.266 (SE 0.196); ^†^Model intercept:-1.320 (SE 0.218); ^††^Model intercept: -6.644 (SE 0.221); ^†††^; Model intercept=-3.150 (SE 0.218).

### 3.2 Disease and mortality of chicken flocks

**Fig. 2:**
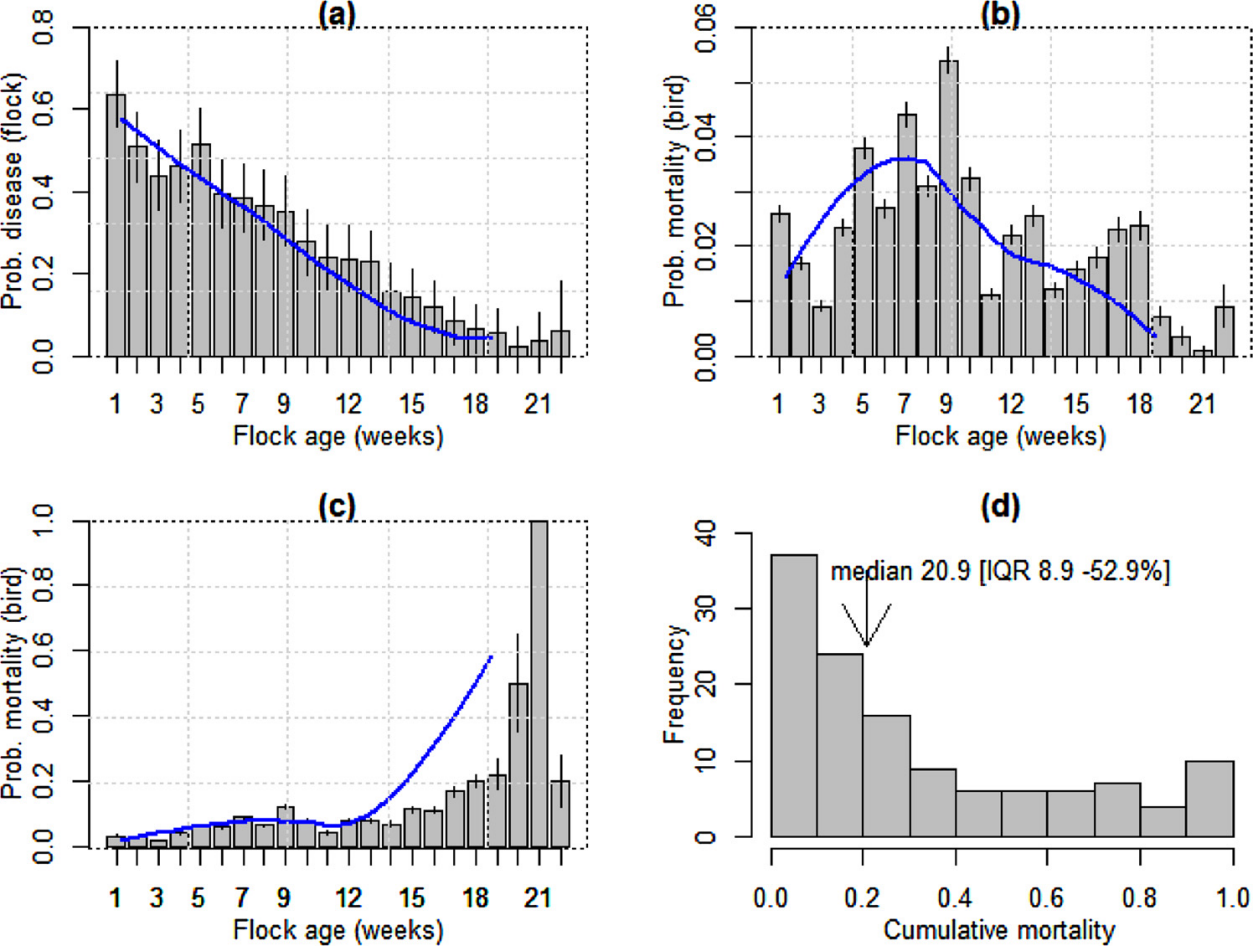
(a) Probability of disease in flocks as a function of their age; (b) Overall weekly incidence of mortality over the observation period; (c) Probability of a bird dying conditional to being in a flock experiencing disease; (d) Frequency distribution of flock cycle (cumulative) incidence of mortality among 124 study flock cycles. The blue lines correspond to a smoothing function fitted by loess regression. (For interpretation of the references to colour in this figure legend, the reader is referred to the web version of this article).

The presence of disease and mortality in a given week in a given flock were highly related (*χ*^2^ = 1780; p < 0.001). The mean weekly incidence of mortality in a given week in a given flock was 0.31 (95% CI 0.29-0.32). The highest probability of disease corresponded to the first week of the cycle (0.64; 95% CI 0.55-0.72), and was inversely correlated with the flocks’ age in weeks (r=−0.95; p < 0.001). After 16 weeks, the probability of disease decreased to <0.1 (Fig. 2a). The (unadjusted) mean weekly incidence of mortality was 0.026 (95% CI 0.022-0.030) (i.e. 2.58 per 100 birds). Mortality was highest during the 5–10 week period, ranging from 0.027 to 0.055 (Fig. 2b). In flocks reporting disease the probability of a bird dying generally increased with the age of the flock (Fig. 2c). The average cumulative mortality over one production cycle was 32.9 per 100 birds (SD ± 30.4), although it was considerably skewed (median 20.9 [IQR 8.9–52.9%]), since there were some flocks where all birds died (Fig. 2d). The most commonly reported clinical signs reported in flocks were, in decreasing order, malaise (weekly probability 0.20; 95% CI 0.19-0.23); diarrhoea (0.06; 95% CI 0.05-0.07); respiratory signs (0.05; 95% CI 0.04-0.06); sudden death (i.e. no prior sign of disease) (0.03; 0.02-0.03), CNS signs (0.01; 95% 0.006–0.014), and lameness (0.01; 95% CI 0.01-0.02) **(**Supplementary Material Figure S1**)**. ‘Other’ disorders included lack of appetite, dehydration, and anaemia. These were reported with a combined probability of 0.09 (95% CI 0.08-0.11). There were differences in the timing of the different conditions: whereas malaise, sudden death, diarrhoea were more often reported in the earlier period, respiratory signs were reported most commonly in weeks 7-13. The weekly incidence of mortality conditional to the presence of respiratory signs was 2.69 (1.27–4.11) for the first 1 to 4 week period, and 9.34 (95% CI 6.15–12.50) for the period spanning from 5 weeks to sale. For weeks reporting diarrhea, the weekly incidence of mortality increased from 4.87 (95% CI 2.91–6.84) (1 to 4 week period) to 13.7 (95% CI 8.98–18.40) (late period).

### 3.3 Use of antimicrobials, vaccines and other health-supporting products

**Fig. 3:**
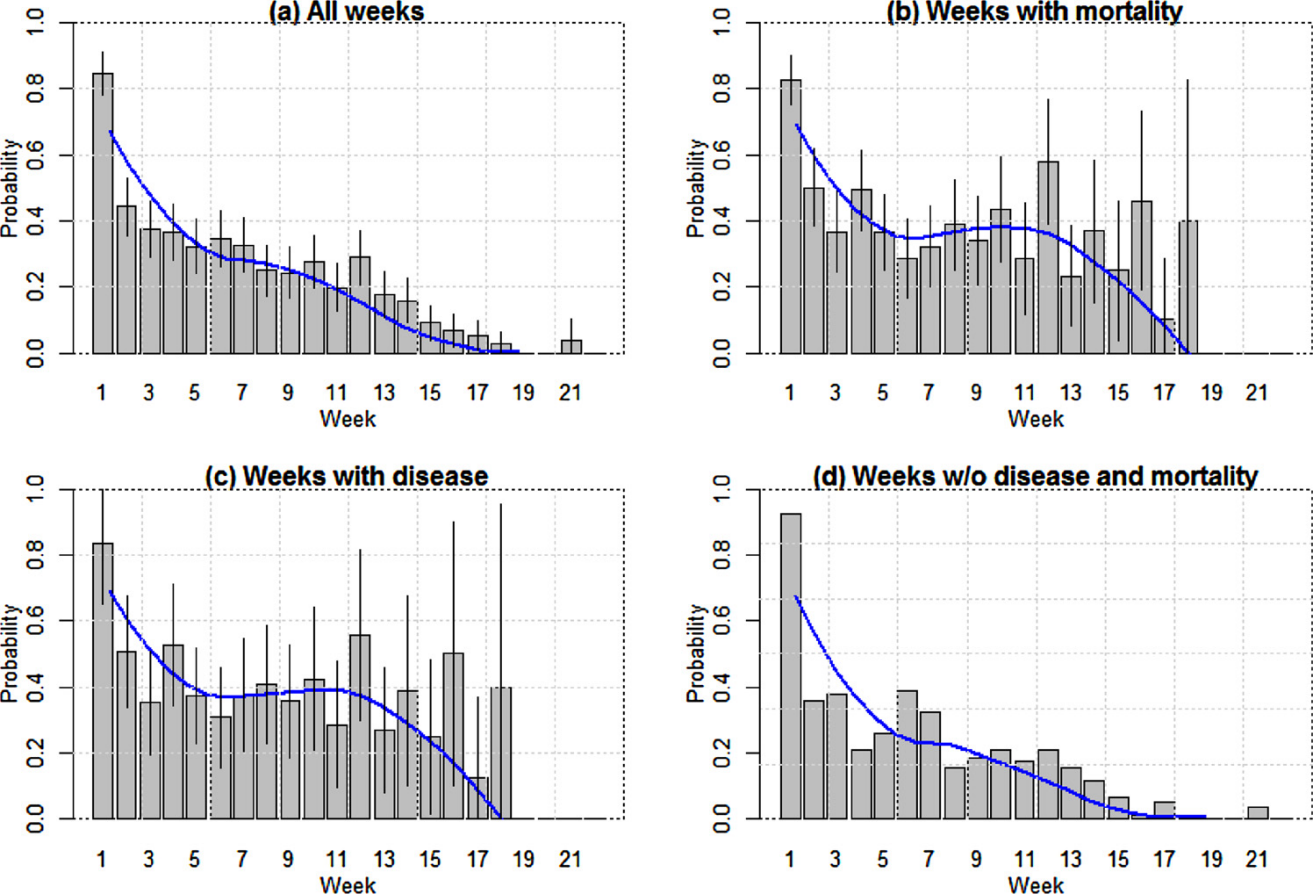
(a) Overall probability of AMU by week; (b) Probability of AMU in weeks with mortality; (c) Probability of AMU in weeks with disease; (d) Probability of AMU in weeks without either disease and mortality (d). The blue lines correspond to a smoothing function fitted by loess regression. (For interpretation of the references to colour in this figure legend, the reader is referred to the web version of this article).

The five most common antimicrobials administered to flocks were colistin (13.9% observation weeks), followed by oxytetracycline (11.4%), tylosin (5.4%), doxycycline (4.4%), and gentamicin (3.0%) (data not shown). Flocks were vaccinated against a median of four different pathogens [IQR 3–4], the most common being Newcastle Disease (91.2% flocks), Highly Pathogenic Avian Influenza (82.4%), Infectious Bursal Disease (Gumboro) (80.0%), Fowlpox (43.2%) and Avian Pasteurellosis (28.0%). The impact of vaccination on disease was not investigated, since vaccines were applied at different times and data on timing of the application was missing in some cases. However, initial analyses did not reveal a significant association between vaccination against specific diseases (Yes/No) and the probability of disease (data not shown). In addition, other non-antimicrobial health-supporting products were used by farmers. These included vitamins/mineral complexes (93.6% flocks), digestive enzymes (77.7%), antiviral products (including interferon) (71.3%), mineral supplements (68.8%), coccidiostats (67.8%), electrolytes (49.5%), and anthelmintics (35.1%). The crude probability of AMU in a given week was 0.26 (95% CI 0.24-0.28) (Fig. 3a). This probability was inversely correlated with the age of the flock (*r*=−0.89; *p* < 0.001). In weeks when disease was reported, the probability of antimicrobial use was 0.43 (95% CI 0.41–0.48) (Fig. 3b, 3c), and 0.18 (95% CI 0.16–0.20) in weeks without disease. There was no difference in the probability of AMU depending on the reported clinical sign (range from 0.43 to 0.49 by clinical sign) (data not shown). There was a weak significant correlation between the probability of AMU in weeks with and without disease in the same flocks (Pearson’s correlation = 0.391, *p* < 0.001) (Supplementary Figure S2). The probability of antimicrobials being used in weeks over subsequent cycles showed moderate correlation (Pearson’s correlation=0.459, *p* < 0.001). This observed correlation was greater than that correlation between the proportion of weeks with disease and/or mortality (Pearson’s correlation=0.040, *p* = 0.8) or average weekly incidence of mortality over two consecutive cycles (Pearson’s correlation=0.108, *p* = 0.5). There were marked differences in the probability of use of antimicrobials between the two study districts (Supplementary Figure S3).

### 3.4 Models of disease, mortality and antimicrobial use

Only the week of production was associated with ‘Disease’ (protective after 5 weeks) (OR ≤ 0.51). Factors independently associated with ‘AMU’ were: (1) No. of chickens (log) (OR = 1.46), (2) Stage of production ≥5 weeks (OR ≤ 0.67) (protective), (3) Cao Lanh district (OR = 2.64), (4) Density of veterinary drug shops at commune level (log) (OR = 1.58), and (5) Disease (OR = 1.80). The variable ‘Density of veterinary drug shops at commune level’, which was not significant in the univariable model, became significant after adjusting by district. Conversely, the variable Density of chickens became non-significant when the variable ‘District’ was added to the model. The variable Mortality become not significant when the variable Disease was introduced. The ICC associated with farm was for models explaining disease/mortality and AMU were 0.288 and 0.226, respectively.

Factors independently associated with overall increased weekly incidence of mortality (*p* < 0.05) were: (1) High level of education attainment (secondary education or higher) (Hazard rate Ratio [HR]=1.70), (2) Number of chickens (log) (HR ≥1.39), and (3) Stage of production >5 weeks (HR≤2.14). In the model using the subset of weeks where farmer reported disease (N=679) with weekly incidence of mortality as the response variable, all three variables fitted in the overall weekly incidence of mortality model remained significant: (1) High level of education attainment (secondary education or higher) (HR=1.58), (2) Number of chickens (log) (protective) (HR=0.89), and (3) Stage of production (HR≥2.87). In addition, AMU remained as a significant (protective) factor (HR=0.90). The two study districts differed in the percent of female farmers: 21.7% (10/46) in Thap Muoi vs. 2.4% (1/42) in Cao Lanh (Fisher’s test, p=0.08). The density of veterinary drug shops by commune in Cao Lanh was also higher than in Thap Muoi (3.1 vs. 1.80 per 10 sq. km) (Kruskal-Wallis *χ*^2^ = 3.46; p = 0.062). Also, the density of chickens in Cao Lanh communes was greater than in Thap Muoi (595.2 vs. 190.6 chickens per km^2^, respectively (Kruskal-Wallis *χ*^2^ = 2.76; p = 0.039). Unlike the variables ‘Density of chickens’ and ‘Density of veterinary drug shops’, the variable ‘Female’ did not remain significant in the AMU model, suggesting that other unmeasured district-associated factors may account for the observed differences.

## 4 Discussion

We characterized disease, mortality, and AMU in small-scale chicken flocks in the Mekong Delta of Vietnam. Although highly variable across all production cycles, the average weekly incidence of mortality was 2.6% (equivalent to a monthly mortality of ˜11%), and the average flock cycle incidence of mortality was ˜33%. We believe that the data collected in this study reflect ‘typical’ farming practices, given that farmers did not receive any advice on husbandry/management practices from the research team. A major limitation of the study lies in the fact that disease status was assessed by farmers, introducing an element of subjectivity, since for some farmers some clinical signs may have appear to be ‘normal’ but not for others, based on their knowledge and experience. In addition, the data on flock disease, mortality and AMU was collected weekly, rather than daily. This did not allow determining in some cases whether the use of antimicrobials precluded the disease onset (prophylactic) or occurred in response to disease (i.e. therapeutic). We believe that, however, the data as a whole represents a valuable source of information on disease and mortality in these small-scale farming systems.

The observed high losses represent a major constraint to the productivity of small-scale systems. This magnitude was considerably higher than that reported from other studies from southern Asia. For example, a study on rural backyard chicken flocks in Cambodia reported average monthly mortalities of 4.5–6.3% (Conan et al., 2013), and a study on scavenging flocks in Bangladesh reported a 2.5% monthly mortality attributable to infectious disease (Biswas et al., 2006). However, in the latter study an additional 2.3% (monthly) mortality due to predation was reported. All our study flocks were penned and often fenced/protected by a mesh during the early brooding period, yet in a few cases chicks were predated by rats in the first few days of life (data not shown). A study from Nigeria reported an average cumulative mortality of 10.4% in small-scale poultry flocks (Muhammad et al., 2010). Our results also indicate a two to three times higher weekly incidence of mortality in these small, commercial farming systems, than in small backyard (median 16 birds [IQR 10–40]) flocks in the Mekong Delta of Vietnam (˜0.75 birds per week) (Delabouglise et al., 2018). There are no comparable data with slow-growing meat chicken flocks. Our observed flock incidence of mortality (˜33%) was also considerably higher than in broiler flocks in Nigeria (12%) (Odemero and Oghenesuvwe, 2016), Norway (2.9% excluding outliers) (Heier et al., 2002) and France (2.7%) (Chauvin et al., 2011).

The probability of disease was highest during the first period of the life of the flock, gradually decreasing thereafter. In contrast, mortality reached a peak during the central 5 to 10-week period, coinciding with the first phase of the ‘grow-out’ period, when chicks are allowed to access to a larger surface of the chicken house, often involving significant changes in feed type and litter conditions. A number of reasons may explain this: (1) waning of maternal and/or vaccinal protective antibodies; (2) increased pathogen challenge in the new environment; and (3) reduced attention paid to the flock by the farmer. Interestingly, it was during the mid-period, when respiratory problems were more often reported. Since diagnostic tests were not performed in our study, it is possible to determine the pathogens responsible for this. Pathogens such as Newcastle Disease virus, HPAI, Infectious Laringotracheitis (ILT) and IBV followed by secondary bacterial infections, or fowl cholera may account for some of this mortality. In addition to HPAI, there is the certainty that Newcastle Disease virus (Choi et al., 2014), and Infectious Bronchitis virus (IBV) (de Witt et al., 2010) are widely circulating in the area (Bui et al., 2001).

Our study confirmed that the presence of disease, rather than mortality, was a key explanatory factor for AMU in small-scale chicken flocks. Older flocks were less likely to be medicated, regardless of the presence of disease. The practice of using antimicrobials to prevent (rather than to treat) disease has been reported previously in chicken farms in the region (Carrique-Mas et al., 2014). Overall, the timing of AMU overlapped well with the presence of disease on farms. In Vietnam a large number of products are marketed as ‘brooding medicine’ (‘thuôc um’), which almost invariably include one or several antimicrobial active ingredients. These products are often supplied by traders together the purchased day-old chicks as a ‘package’ (See Supplementary Figure S4 for a description of four representative products). Day-old chicks are typically brought to the farm by traders on motorbike, often involving travelling for over 100 km under a hot and humid climate, often resulting in poor condition of birds on arrival. Hatchery sources have been found to be associated with mortality in a number of studies (Heier et al., 2002; Muhammad et al., 2010). The data clearly showed that farmers tend to repeat their antimicrobial use patterns over subsequent cycles. Surprisingly, we found that in about ˜50% of weeks where flocks had overt signs of disease farmers did not administer antimicrobials. This occurred in situations when farmers judged the disease episode as mild, or in situations when farmers administered non-antimicrobial medicinal products such as vitamin complexes, minerals, enzymes, antibodies, and interferon (against suspected viral infections). We found that larger flocks had generally increased mortality and increased AMU levels. This contrasts with previous findings from a survey of poultry farms in a different province in the Mekong Delta, where smaller farms were at increased risk of AMU (Carrique-Mas et al., 2014). However, in that study smaller flocks were mostly backyard flocks, whereas all our study flocks were confined and single age. Interestingly, the density of veterinary drug shops was positively associated with increased AMU (OR = 1.58), suggesting that the availability of antimicrobials in veterinary drug shops may be a driving factor for AMU. In a previous study in another province in the Mekong Delta the veterinary drug shop was cited by 56% chicken farmers as their main source of procurement and advice of antimicrobial drugs to the farmers (Carrique-Mas et al., 2014). The differences observed between districts may also respond to differences in purchasing power of farmers these two districts. In addition, Cao Lanh district is closer to the provincial capital, with many more veterinary drug shops within close range. We have no explanation for the higher levels of mortality in flocks owned by farmers with higher education attainment. We did not find that this association was confounded by experience, district or any other variable. A possible explanation for this is that education is a proxy of wealth, and wealthier farmers have a wider range of occupations, and may therefore be less committed to tending their flocks. Given the presence of disease in the flock, the use of antimicrobials resulted in significantly lower weekly incidence of mortality (HR = 0.90), suggesting that therapeutic use of antimicrobials somehow reduces losses due to disease, although the magnitude of the observed reduction is small.

Our study focused on non-intensive, commercial chicken farms. Non-industrial farming production still account for the majority (60%) of chicken production in Vietnam (65% in the Mekong Delta region) (VCNST, 2018). The fragmentation of the Vietnamese farming landscape and the country’s dependence on imported animal feeds, represent a major constraint to large-scale industrialization of poultry production (Ipsos Business Consulting, 2018). In addition, the Vietnamese consumer has a predilection for traditional, slow-growing breeds due to improved taste and texture. However the prolonged raising period required for these breeds represents an additional risk of disease introduction (Hong Hanh et al., 2007a).

## 5 Conclusions

We report exceptionally high levels of mortality in small-scale chicken flocks based on slow-growing breeds, and a clear association between the early brooding phase and the presence of disease and/or mortality and AMU in flocks. In addition, the link between AMU and the density of veterinary drug shops at commune level, as well as other unidentified district-related factors, suggest that the market availability of antimicrobials and other cultural factors may contribute to explain AMU on farms. The study also highlights the benefits of regular (ideally daily) data collection on disease and mortality at farm-level, and therefore we encourage producers in the area to follow this practice. The results strongly suggest that farmers need to focus their efforts on controlling disease and mortality during the first 10 weeks of the life of the flock, improving chicken house sanitation and stepping up biosecurity to reduce the risk of introduction of disease. The presence of large numbers of small-scale chicken farms presents major challenges to the development of policies aimed at AMU reductions. We recommend that these policies include the stewardship of the antimicrobial use in farming systems in the region.
