# Supplementary material for: Mortality, disease and associated antimicrobial use in commercial small-scale chicken flocks in the Mekong Delta of Vietnam
Source: Prev Vet Med. 2019 Apr 1;165:15–22. doi: 10.1016/j.prevetmed.2019.02.005 (PMC6418316; doi:10.1016/j.prevetmed.2019.02.005)
Supplement: Supplementary file 1 [file mmc1.docx]

**Supplementary Figure S1.** Probability of reporting clinical signs by week over 124 cycles of production. The blue lines correspond to a smoothed polynomial smoothing function fitted by loess regression.


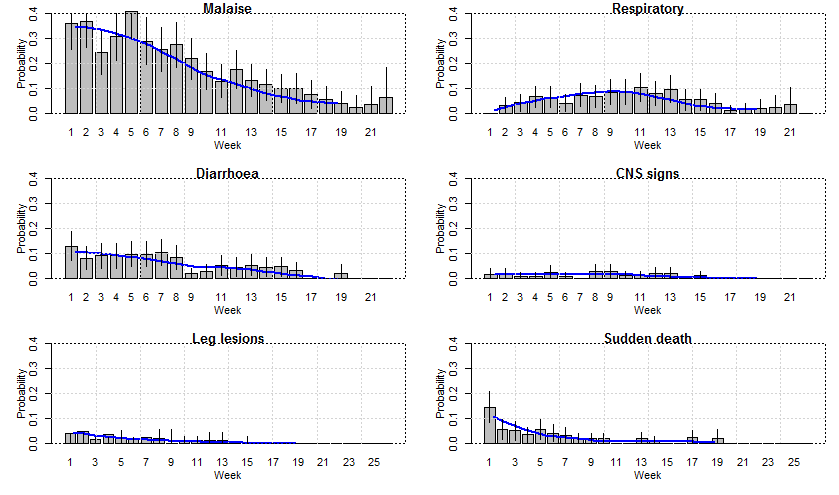


**Supplementary Figure S2.** (a) Relationship between the probability of using antimicrobials in weeks where there is and there is no disease in each flock cycle; (b) Relationship between the probability of disease and/or mortality over subsequent cycles; (c) Relationship between the weekly incidence of mortality (per 100 birds) over two adjacent cycles; (d) Relationship between the probability of AMU over subsequent cycles of production.


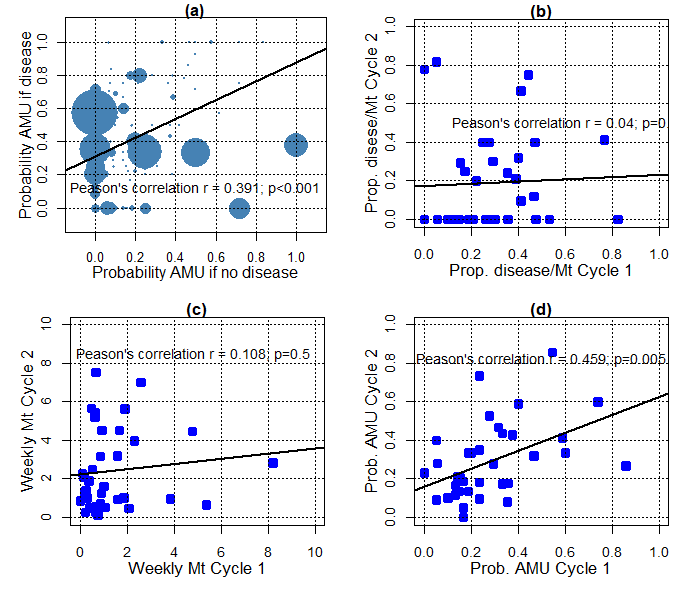


**Supplementary Figure S3.** Comparison of AMU in flocks Thap Muoi and Cao Lanh districts. The blue lines correspond to a smoothed polynomial smoothing function fitted by loess regression.


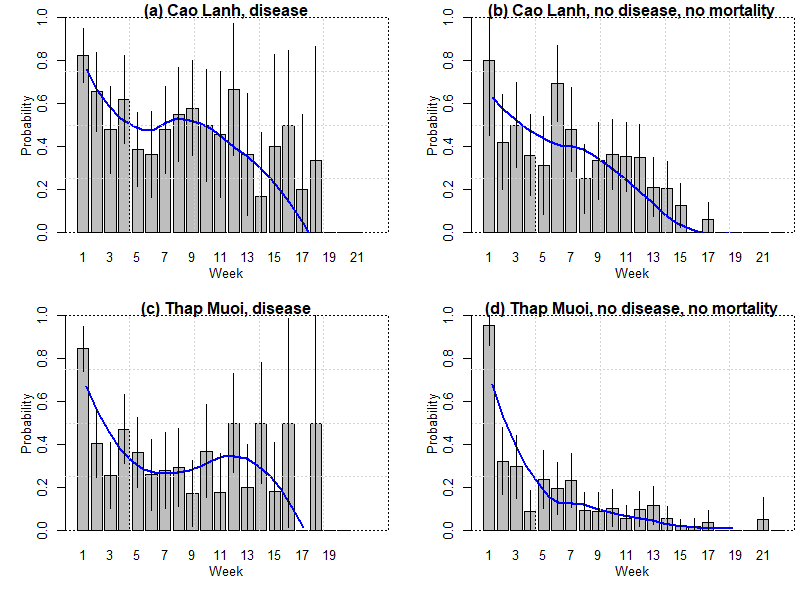


| Plate 1: **UM GA VIT** (“Brooding product for chickens and ducks). Antimicrobial mix containing spiramycin and colistin; indications are the prevention and treatment of disease. | Plate 1: **UM GA. VIT, NGAN** (“Brooding product for chickens, ducks and Muscovy duck). Product containing vitamins and aminoacids (unspecified) and amoxicillin; indications are the prevention and treatment of disease. |
| --- | --- |
| 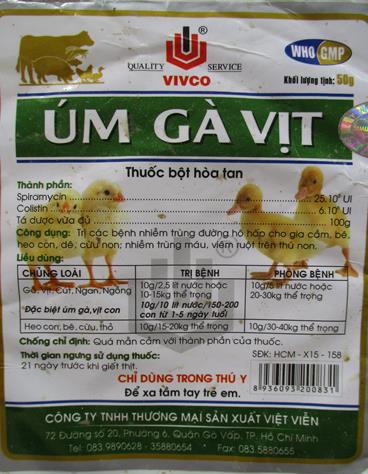 | 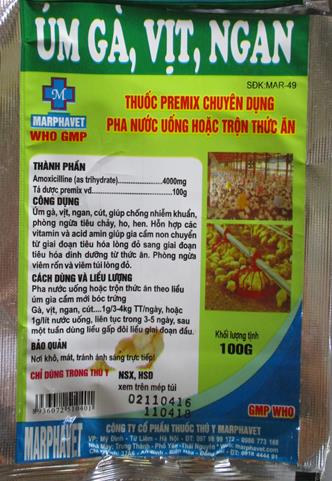 |
| Plate 3: **NANO UM** (“Brooding product for chicken, ducks and Muscovy ducks”) Multivitamin/Aminoacid/Calcium complex containing oxytetracycline. | Plate 4: **UM GIA CAM** (“Brooding product for poultry”). Multivitamin/Aminoacid/Calcium complex containing erythromycin. |
| 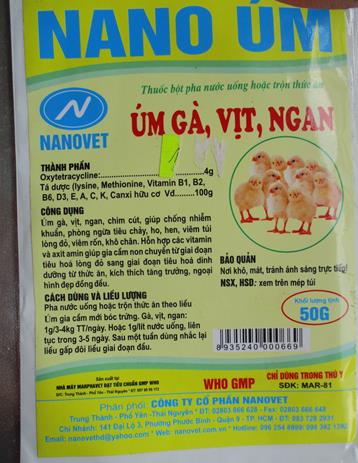 | 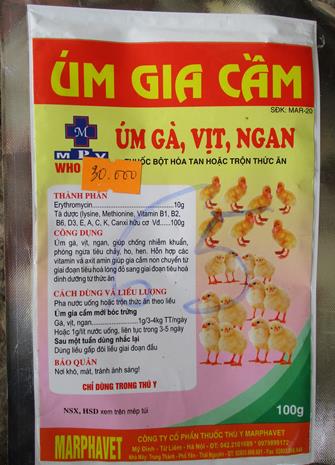 |

**Supplementary Figure S4.** Representative products purchased by chicken farmers in the Vietnamese Mekong Delta aimed at improving the health during the brooding period.
